# Daily calcium intake in male children and adolescents obtained from the rapid assessment method and the 24-hour recall method

**DOI:** 10.1186/1475-2891-6-24

**Published:** 2007-09-19

**Authors:** Michael Moore, Sarah Braid, Bareket Falk, Panagiota Klentrou

**Affiliations:** 1 Faculty of Applied Health Sciences, Brock University, Ontario, Canada

## Abstract

**Background:**

In order to rapidly assess nutrient intake, Food Frequency Questionnaires (FFQ) have been developed and proven to be reliable for quick, user friendly analysis in adults. However, the accuracy of these questionnaires in children has been studied to a limited extent. The aim of this study was to compare the daily calcium intake values obtained from the Rapid Assessment Method (RAM), an FFQ, for assessing daily calcium intake in child and adolescent males with the values obtained from the 24-hour recall method.

**Methods:**

Subjects included 162 child and adolescent males, aged 9–16 years, subdivided into elementary school (ES, 9–12 years) and high school (HS, 14–16 years) age groups.

**Results:**

Daily calcium intake was significantly lower in ES compared with HS, using both methods. The intra-class correlation coefficients (ICC) between RAM values and those obtained using the 24-hour recall questionnaire were significant yet moderate (ICC = 0.46 and 0.43 for ES and HS, respectively). However, daily calcium intake obtained using RAM was significantly higher when compared with the 24-hour recall values in both ES (1576 +/- 1101 vs. 1003 +/- 543 mg, in RAM and 24-hour, respectively) and in HS males (1873 +/- 739 vs. 1159 +/- 515 mg, in RAM and 24-hour, respectively).

**Conclusion:**

RAM overestimates daily calcium intake as compared with the 24-hour recall method in both child and adolescent males.

## Background

Low levels of calcium intake are becoming increasingly prevalent among children and adolescents [1]. This has been suggested to impede the attainment of peak bone mass during adolescence [2]. Thus, there is an imminent need for a valid and efficient calcium assessment tool in this population. Food consumption data are collected using a wide variety of methods and procedures [3]. The 24-hour recall method is the most commonly used assessment tool in large cross-sectional surveys and skeletal development studies in both children and adults. This method has numerous advantages including responsiveness to change in food supply and habit [3,4]. The advantage of 24-hour recalls is that employs probes and checks to ensure that responses are correct and accurate. In large samples, the 24-hour recall is said to provide valuable information on differences between group averages [5,6]. Although the 24-hour recall method may yield variable responses in young children, Livingstone and Robson [7] found that from 8 years of age, there is a rapid increase in the ability of children to report food intake. Moreover, Baranowski et al. [8] found strong agreement (82% for all foods) between children's self-reported frequencies and meal observations.

Although the 24-hour recall method has proven to be effective in older children, it does require analysis through dietary software, and attaining specific nutrient values (such as calcium) can be time-involved. Studies that are focused on bone growth and development require a tool that will quickly diagnose nutrient intake of calcium in children and adolescents. In order to assess nutrient intake in a rapid fashion, Food Frequency Questionnaires (FFQ) have been developed. In assessing calcium intake, one of the more commonly used FFQs in clinical research is the Rapid Assessment Method (RAM). In adults, RAM has been found to be reliable and valid [9,10]. Ward et al. [10] 2004 found that 84% of college athletes with an inadequate calcium intake (based on 6-day dietary recalls) were quickly and accurately identified though the administration of the calcium RAM. However, in spite of the importance of calcium intake during the growing years, RAM has never been used or validated in children and adolescents.

As previously stated, the 24-hour recall method is the most often used dietary assessment tool in large clinical studies. This method has often been used with children and adolescents [11-14]. However, its use is time-consuming and its analysis often requires expertise and special software. Single 24-hour dietary recalls are advantageous in clinical use for this population because they provide checks and time references (all foods listed and accounted for) for the child in a capacity that they can comprehend [5]. FFQs on the other hand, can be time efficient and are readily available. Thus, the aim of this study was to compare the daily calcium intake values obtained from the Rapid Assessment Method (RAM), an FFQ, for assessing daily calcium intake in child and adolescent males against the values obtained from the 24-hour recall method.

## Methods

One hundred and sixty-two children aged 9–16 years from Southern Ontario, Canada participated in the study. Subjects were recruited from numerous schools and sports clubs. As this project is part of a larger study, only male subjects were recruited at this point. All testing was approved by the Brock University Research Ethics Board. Informed written consent was obtained from both the subjects and their parents/guardians prior to their involvement with the study. Subjects were divided into one hundred and seven (107) elementary school (ES, 9–12 years, mean age 11.2 ± 0.7 years) and fifty-five (55) high school (HS, 14–16 years, mean age 15.4 ± 0.5 years) groups. These two groups were selected to represent two different levels of pubertal maturity. Indeed, pubertal maturity, as self-assessed using secondary sexual characteristics [15,16], was lower in ES (Tanner stages 1–2) as compared with HS (Tanner stages 4–5). Body weight was measured using a calibrated Zenith digital scale. Height was measured with an Ellard Instrumentation board length statiometer (Monroe, WA, U.S.A). Relative body fat was estimated from skinfold thicknesses of the triceps and subscapular landmarks [17]. As expected, the groups were significantly (*p *< 0.05) different in terms of physical characteristics with ES boys being smaller in size (41.2 ± 10.2 kg, 146.6 ± 7.8 cm) than HS (65.3 ± 11.1 kg, 172.6 ± 7.0 cm). Both groups had a relatively small percentage of boys that were overweight (23% and 13% for ES and HS, respectively) with no significant differences in relative body fat between groups (19.3% and 16.4% body fat for ES and HS, respectively). Additionally, no boys that were classified as obese were used in the analysis of this study.

Each subject attended a nutritional interview in a private room. The 20- to 30-minute interview included the calcium RAM [9] and a 24-hour dietary recall. Both questionnaires were administered using visuals aids to approximate the serving sizes of various foods. Subjects report was taken independent of parental response, as parents have been found to not be reliable reporters of their children's food intake out-of-home [8]. All interviews were conducted by the same trained and experienced researcher.

The RAM includes 30 items and refers to a typical day [see Additional file 1]. Two categories were added to the original RAM questionnaire, namely calcium enriched orange juice and specific nutritional supplements. The calcium values (mg) for nutritional supplements and calcium enriched orange juice were obtained through the Diet Analysis 6.0 program (Belmont, CA, U.S.A).

For the 24-hour dietary recall, subjects were asked to recall everything consumed (including foods, beverages, sauces and condiments) the day prior to the interview. Prior to answering the 24-hour dietary recall, subjects were asked if the last 24 hours were typical for their diet. If it was not a *typical *day (e.g. birthday party, family gathering, eating out), they reported two days prior to the interview date. The 24-hour dietary recall started from the first meal or beverage consumed at waking until midnight of the reporting day. The data were analyzed using the Diet Analysis 6.0 program for total energy intake, and total calcium intake. When nutritional information was not available through the software program, manufacturer's labels and information from fast food chains were utilized.

### Statistical Analysis

Intra-class correlation was used for the comparison between the RAM and the 24-hour recall method. A one-way ANOVA was used to determine differences in anthropometric variables between groups. A paired t-test was used to compare the mean daily calcium intake obtained by RAM with that calculated based on the 24-hour dietary recall. To examine age differences in the validity of RAM, an ANOVA for repeated measures, with age-group (ES vs. HS) being the between-subject factor and assessment method (RAM vs. 24-hour) being the within-subject factor, was also performed. All data are expressed as mean ± SD. Statistical significance was accepted at *p *≤ 0.05.

## Results

**Figure 1:**
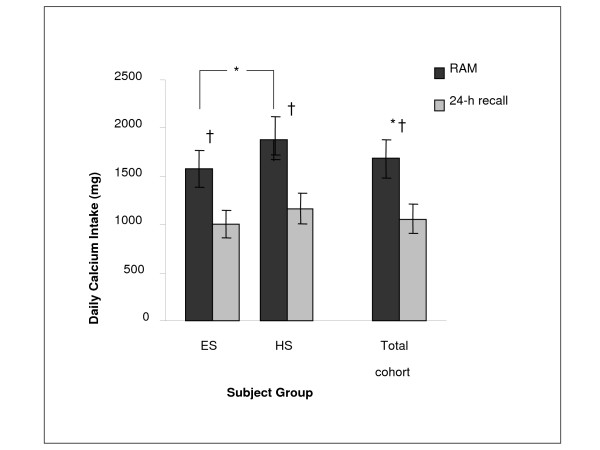
Daily calcium intake for both age groups obtained from RAM and the 24-hr recall (mean ± SD; ES = elementary school age; HS – high school age; * *p *≤ 0.05 between age-groups; † *p *≤ 0.05 between methods).

The intra-class correlation coefficients (*ICC*) between RAM daily calcium intake values and those obtained using the 24-hour recall questionnaire were significant (*p *< 0.05) yet moderate (*ICC *= 0.46 and 0.43 for ES and HS, respectively). However, daily calcium intake obtained using RAM was significantly higher when compared with the 24-hour recall values, with a similar pattern being observed in each age-group separately (Figure 1). Calcium intake (Figure 1), and total daily energy intake (8225.7 ± 3087.8 vs. 10175.5 ± 3351.4 kJ in ES and HS, respectively) were significantly higher in HS.

## Discussion

This is the first study that attempted to compare the use of this specific FFQ against the 24-hour recall questionnaire in the nutritional assessment of male children and adolescents. It was found that the RAM consistently overestimated daily calcium intake compared with the 24-hour recall, suggesting that this method of nutritional assessment should be used with caution in these age groups.

Studies investigating the differences in calcium intake between FFQ and dietary records or recalls in children are limited. Similar to our finding, another FFQ, the Block98, was found to overestimate energy and macronutrient intake when compared with a 3-day dietary record in 4–9 year-old girls [18]. Recently, Bertoli et al. [19] reported that another FFQ overestimated calcium intake when compared with a 7-day weighed record diary in 6–20 year old Italians. Since dietary recalls of more than 1-day have been reported to be problematic for younger children [8], the present study compared the RAM with a 24-hour dietary recall. A number of recent studies have also used 24-hour recalls to validate different calcium FFQs [20-22]. In accordance with our results, Harnack et al. [21] also reported that although the test-retest reliability of their 10-item calcium FFQ was good (*ICC *= 0.66), the calcium intake values from FFQ was only moderately associated with estimates from 24-hour recalls (*ICC *= 0.40) in 11–14 years old boys. Magkos et al. [22] found that their 30-item calcium FFQ significantly underestimated the calcium intake in 351 Greek children aged 11.9 ± 1.2 years. The latter study is in contrast with our findings of overestimation of calcium intake in children and adolescents by the RAM. The discrepancy may be due to the different FFQs used or to the different age groups of the subjects. Nevertheless, our results strengthen previous reports of FFQs' overestimation of nutrient intake in children.

We suggest that the overestimation of FFQs may be attributed to their typically long list of food items. The abundant choices may make the children feel obliged to mark more food items than they actually consumed, thereby over-reporting calcium intake. On the other hand, it is possible that 24-hour recall underestimated calcium intake of our subjects. Using the mean values for height, weight, and age of our participants, and assuming low to high physical activity levels, mean energy expenditures for the ES boys would be approximately 8995 and 10376 kJ/d, respectively, whereas requirements for HS boys would be approximately 12133 and 13807 kJ/d, respectively (calculated using the 2005 EER equations from the Institute of Medicine) [23]. Thus, it appears that the 24-hour recall likely underestimated total energy intake by about 10% (ES, assuming low activity) to as much as 35% (HS, assuming high activity). While it cannot be assumed that calcium intake would be proportionally underestimated, it is possible that the 24-hour recall method may have slightly underestimated daily calcium intake. Moreover, the daily calcium values obtained (Figure 1) from the RAM, for both ES and HS groups, appear to be in contrast and overestimated as compared with past research utilizing multi-pass 24-hour recalls or 6 day dietary records [5,24].

## Conclusion

The problem of estimating the proportion of the population at risk for dietary inadequacy has yet not been resolved. In our study, it is concluded that the RAM overestimated daily calcium intake as compared with the 24-hour recall method in both child and adolescent males. The growing years have been called the 'window of opportunity' for preventing degenerative bone diseases, such as osteoporosis, in the future [25]. It is crucial that researchers be able to attain accurate calcium values in children and adolescents to ensure proper bone accrual during the growth years. Further studies are required to identify a valid tool for assessing calcium intake in male and female children and adolescents.

## Competing interests

The author(s) declare that they have no competing interests.

## Authors' contributions

MM administered the nutritional questionnaires and interviews, performed the analysis of the nutritional questionnaires, determined all skinfold measures, assisted with subject recruitment, and drafted the manuscript. SB was responsible for subject recruitment, performed anthropometric measurements and assisted in the nutritional analysis. BF conceived of the study, and participated in its design and coordination. PK designed and supervised the study, performed the statistical analysis and helped to draft the manuscript. All authors contributed to the writing of the final manuscript.

## Supplementary Material

Additional file 1 Calcium Rapid Assessment Method. The RAM food frequency questionnaire Click here for file

## References

[B1] Cavadini C, Decarli B, Grin J, Narring F, Michaud PA (2000). Food Habits and sport activity during adolescence: differences between athletic and non-athletic teenagers in Switzerland. Eur J Clin Nutr.

[B2] O' Dea J (2003). Calcium, growth and health in children and adolescents. Nutridate.

[B3] Guenther PM, Kott PS, Carriquiry AL (1997). Development of an approach for estimating usual nutrient intake distributions at the population level. J Nutr.

[B4] Harrison GG, Galal OM, Ibrahim N, Khorshid A, Stormer A, Leslie J, Taha Saleh N (2000). Underreporting of food intake by dietary recall is not universal: A comparison of data from Egyptian and American women. J Nutr.

[B5] Field AE, Peterson KE, Gortmaker SL, Cheung L, Rockett H, Fox MK, Colditz (1999). Reproducibility and validity of a food frequency questionnaire among fourth to seventh grade inner-city school children: Implications of age and day-to-day variation in dietary intake. Public Health Nutrition.

[B6] Rockett HRH, Colditz GA (1997). Assessing diets of children and adolescents. Am J Clin Nutr.

[B7] Livingstone MBE, Robson PJ (2000). Measurement of dietary intake in children. Proceedings of the Nutritional Society.

[B8] Baranowski T, Dworkin R, Henske JC, Clearman DR, Dunn JK, Nader PR, Hooks PC (1986). The accuracy of children's self reports of diet: Family Health Project. J Am Diet Assoc.

[B9] Hertzler A, Frary R (1994). A dietary calcium rapid assessment method (RAM). Top Clin Nutr.

[B10] Ward KD, Hunt KM, Berg MB, Slawson DA, Vukadinovich CM, McClanahan BS, Clemens LH (2004). Reliability and validity of a brief questionnaire to assess calcium intake in female collegiate athletes. Int J Sport Nutr Exerc Metab.

[B11] Reynolds K, Baranowski T, Bishop D, Farris R, Binkley D, Nicklas T, Elmer P (1999). Patterns in Child and Adolescent Consumption of Fruit and Vegetables: Effects of Gender and Ethnicity across Four Sites. J Am Coll Nutr.

[B12] Falk B, Bronshtein Z, Zigel L, Constantini N, Eliakim A (2004). Higher tibial quantitative ultrasound in young female swimmers. Br J Sports Med.

[B13] Falk B, Galili Y, Zigel L, Constantini N, Eliakim A (2007). A Cumulative Effect of Physical Training on Bone Strength in Males. Int J Sports Med.

[B14] Millen A, Midthune D, Thompson F, Kipnis V, Subar A (2006). The National Cancer Institute Diet History Questionnaire: Validation of Pyramid Food Servings. Am J Epidemiol.

[B15] Tanner JM (1962). Growth at Adolescence (2nd edition).

[B16] Taylor S, Whincup P, Hindmarsh P, Lampe F, Odoki K, Cook D (2001). Performance of a new pubertal self-assessment questionnaire: A preliminary study. Paediatric Perinatal Epidemiol.

[B17] Slaughter MH, Lohman TG, Boileau BA (1988). Skinfold equations for estimation of body fatness in children and youth. Hum Biol.

[B18] Wilson A, Lewis R (2004). Disagreement of energy and macronutrient intakes estimated from a food frequency questionnaire and 3-day diet record in girls 4 to 9 years of age. J Am Diet Assoc.

[B19] Bertoli S, Petroni ML, Pagliato E, Mora S, Weber G, Chiumello G, Testolin G, Bertoli (2005). Validation of food frequency questionnaire for assessing dietary macronutrients and calcium intake in Italian children and adolescents. J Pediatr Gastroenterol Nutr.

[B20] Jensen JK, Gustafson D, Boushey CJ, Auld G, Bock MA, Bruhn CM, Gabel K, Misner S, Novotny R, Peck L, Read M (2004). Development of a food frequency questionnaire to estimate calcium intake of Asian, Hispanic and white youth. J Am Diet Assoc.

[B21] Harnack LJ, Lytle LA, Story M, Galuska DA, Schmitz K, Jacobs DR, Gao S (2006). Reliability and validity of a brief questionnaire to assess calcium intake of middle-school-aged children. J Am Diet Assoc.

[B22] Magkos F, Manios Y, Babaroutsi E, Sidossis LS (2006). Differences in quantitative and qualitative performance of a calcium-specific food frequency questionnaire across age and sex. J Hum Nutr Diet.

[B23] Institute of Medicine (2005). Dietary Reference Intakes for energy, carbohydrate, fiber, fat, fatty acids, cholesterol, protein, and amino acids (macronutrients).

[B24] Ortega RM, Requejo AM, López-Sobaler AM, Andrés P, Quintas ME, Navia, Izquierdo M, Rivas T (1998). The importance of breakfast in meeting daily recommended calcium intake in a group of school children. J Am Coll Nut.

[B25] MacKelvie KJ, Khan KM, McKay HA (2002). Is there a critical period for bone response to weight-bearing exercise in children and adolescents? A systematic review. British J Sports Med.

