# Supplementary material for: Daily calcium intake in male children and adolescents obtained from the rapid assessment method and the 24-hour recall method
Source: Nutr J. 2007 Sep 19;6:24. doi: 10.1186/1475-2891-6-24 (PMC2075497; doi:10.1186/1475-2891-6-24)
Supplement: Additional file 1 — Calcium Rapid Assessment Method. The RAM food frequency questionnaire [file 1475-2891-6-24-S1.pdf]

The Calcium Rapid Assessment Method

FOOD FREQUENCY QUESTIONNAIRE

Record the number of servings you ate on a typical day in the previous 7 days.
(Use the pictures to estimate serving sizes)

MILK -YOGURT-CHEESE	# SERVINGS DAILY
Cheese, 1oz or 6 tbsp.	
Cottage cheese, ½ cup	
Custard, pudding, or cream pie, ½ cup	
Ice cream, frozen yogurt, or milk shake, 1 cup	
Milk or cocoa, 1 cup	
Soy milk, 1 cup	
Yogurt, 1 cup	
Cream soups/sauce, 1 cup	
Macaroni and cheese, 1cup	
Pizza, 1/8 of 15″ (8 slice pizza)	
Quiche, 1/8 of 8″	

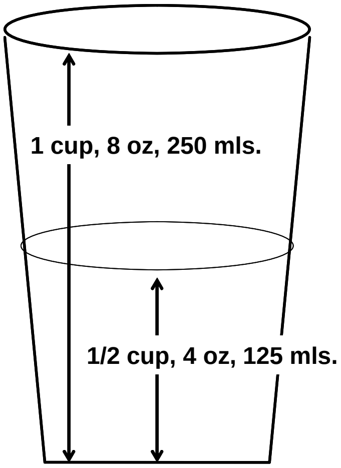

FRUITS and VEGETABLES	# SERVINGS DAILY
Broccoli or cooked greens (beet/turnip greens, kale, collards, spinach), ½ cup	
Other vegetables, ½ cup	
Orange juice, 1 cup (enriched with calcium)	
Fruits, ½ cup or 1 small	

MEAL REPLACEMENT PRODUCTS	# SERVINGS DAILY
Slim fast, 1 can	
Jenny Craig bar, 1 bar	
Other:____________________________	

- **Fist = 1 cup or 1 medium whole fruit**
- **Thumb (tip to base) = 1 oz. of meat or cheese**
- **Thumb tip (tip to 1st joint) = tbsp.**
- **Index finger (1st to 2nd joint) = 1″**
- **Palm (minus fingers = 3 oz. of meat, fish, or poultry**

BREADS-CEREALS-RICE-PASTA	# SERVINGS DAILY
Bread, 1 slice	
Bread, 1 slice (enriched with calcium)	
Cereal, 1 oz	
Cereal, 1 oz (enriched with calcium)	
2″ biscuit/roll	
6″ corn tortilla	
3″ muffin, cornbread, or doughnut	
Rice, noodles, or pasta, 1 cup	
Pancake, waffle, or French toast, 1 serving	

FAT-SUGAR-ALCOHOL	# SERVINGS DAILY
Cake, 1/16 of 9″	
Beer, 12oz	
Colas, 12oz	
Chocolate, 1oz	
**MEAT-FISH-POULTRY-DRY BEANS-NUTS**	**# SERVINGS DAILY**
Dry beans, cooked (navy, pinto, kidney), 1 cup	
Meat, fish, poultry, 3 oz	
Peanuts, ½ cup	
Almonds, ½ cup	
1 egg	
Salmon (with bones), 3oz	
Sardine (with bones), 3 oz	
3oz shrimp	
7 to 9 oysters	
Tofu, 2 ½″x 2 ½″x 1″	
